# Cartilage repair mediated by thermosensitive photocrosslinkable TGFβ1-loaded GM-HPCH via immunomodulating macrophages, recruiting MSCs and promoting chondrogenesis

**DOI:** 10.7150/thno.41622

**Published:** 2020-02-03

**Authors:** Xiongfa Ji, Zehua Lei, Meng Yuan, Hao Zhu, Xi Yuan, Wenbin Liu, Hongxu Pu, Jiawei Jiang, Yu Zhang, Xulin Jiang, Jun Xiao

**Affiliations:** 1 Department of Orthopaedic Surgery, Tongji Hospital, Tongji Medical College, Huazhong University of Science and Technology, Wuhan, 430030, China; 2 Key Laboratory of Biomedical Polymers of Ministry of Education & Department of Chemistry, Wuhan University, Wuhan, 430072, China.; 3 Department of Orthopedics, Guangdong General Hospital, Guangdong Academy of Medical Sciences, Guangzhou, Guangdong 510080, PR China

**Keywords:** thermosensitive photocrosslinkable hydrogel, mechanical improvement, TGFβ1, immunomodulation, cell recruitment, cartilage tissue engineering

## Abstract

Repairing cartilage defects using thermosensitive hydrogels is an attractive treatment strategy, but the poor mechanical properties and limited understanding of the interactions between hydrogels and cells limit their application.

**Methods**: In this study, a thermosensitive hydroxypropyl chitin hydrogel (HPCH) was functionalized with methacrylate groups to synthesize photocrosslinkable glycidyl methacrylate-modified HPCH (GM-HPCH). GM-HPCH could form a gel *in situ* through a thermosensitive sol-gel transition and its mechanical properties can be improved by UV irradiation. Cell viability, cell adhesion and anti-apoptosis activity of GM-HPCH were evaluated. Transforming growth factor-β1 (TGFβ1) was introduced into the GM-HPCH hydrogel to fabricate the composite hydrogel. The macrophage immunomodulation, MSC recruitment and chondrogenesis of the composite hydrogel were evaluated.

**Results**: With high biocompatibility, GM-HPCH could protect chondrocytes from apoptosis. Both the *in vitro* and *in vivo* experiments showed that GM-HPCH + TGFβ1 shifted the recruited macrophages from M1 to M2 and promoted chondrogenic gene expression. Additionally, the composite hydrogel could promote the migration of marrow stromal cells (MSCs) in the Transwell test and increase migrated gene expression. The fluorescent tracking of MSCs confirmed MSC homing in the rat chondral defect with the help of GM-HPCH. The macroscopic evaluation and histological results at 6 weeks and 12 weeks postsurgery showed that GM-HPCH + TGFβ1 can achieve superior cartilage healing.

**Conclusions**: The GM-HPCH + TGFβ1 hydrogel effectively promoted cartilage repair via immunomodulating macrophages, recruiting MSCs and promoting chondrogenesis; thus it is a promising injectable hydrogel for cartilage regeneration.

## Introduction

Articular cartilage is a viscoelastic tissue with a highly organized structure 1. Cartilage defects caused by trauma, tumors, or osteoarthritis are challenging to repair because of the limited intrinsic potential for self-healing 2. Traditional clinical therapies 3, including microfractures, cartilage allografts, arthroplasty, and injection of bone marrow stem cells (MSCs), have yielded limited results. In contrast, hydrogel-based tissue engineering is a promising strategy due to its simplicity and stable drug-carrying ability, thus promoting cartilage regeneration.

The crosslinking pattern of a hydrogel can influence its applications. Chemical crosslinking can obtain high-quality gelation. Photosensitive hydrogels are attractive chemical crosslinking hydrogels; their improved mechanical properties underlie their suitability for application to cartilage tissue engineering because articular cartilage is load-bearing 4. However, it is a time-consuming process and cannot yield immediate gelation. Hydrogels quickly diffuse from the target site before gelation, whereas physically crosslinked hydrogels, such as thermosensitive hydrogels, gel more quickly *in situ* once the temperature changes 5. Thermosensitive hydrogels 6, such as Chitosan 7, poly(N-isopropylacrylamide) (PNIPAAm) 8, pluronic F127 9, and methacrylated hyaluronic acid (HAMA) 10, have been used as cartilage-repairing hydrogels, and cartilage-related matrix have been detected at the defect site. However, the mechanical properties of physically crosslinked hydrogels are weaker than those of chemically crosslinked hydrogels. It is difficult to maintain their shape when implanting *in vivo*, and weight is easily lost; this limits their applications. Although the implanted hydrogel acts as a temporary material to support cartilage regeneration, it is crucial to maintain the original shape and to guide the chondrocytes to attach to and secrete into the matrix. Thus, it is essential to functionalize hydrogels via both physical and chemical crosslinking. In our previous work, we synthesized a thermosensitive hydroxypropyl chitin hydrogel (HPCH). The hydroxyl groups of chitin were etherified with propylene oxide in NaOH/urea solution, which is regarded as a “green” solvent. The hydrogel has been demonstrated to have good biocompatibility and biodegradability. Glycidyl methacrylate was used to functionalize HPCH with photocrosslinkable methacrylate groups to improve its mechanical properties. The compress modulus of glycidyl methacrylate-modified HPCH (GM-HPCH) after UV exposure was nine times better than that of the original HPCH or GM-HPCH before photocrosslinking 11. GM-HPCH can quickly form a gel, thus maintaining its shape when filling an irregular cavity. Photocrosslinking improves the mechanical properties of the hydrogel to resist stress and weight loss. This novel thermosensitive and photocrosslinkable hydrogel is thought to be suitable for cartilage tissue engineering.

The activation of macrophage systems is an essential process during biomaterial implantation and cartilage regeneration. During the early stages of repair, the macrophage activation type is M1, acting as the “cleaner” by means of promoting inflammation. As the repair process progresses, the M1 macrophages change to M2 macrophages, which are regarded as regenerative homeostasis promoters 12. The interactions between implanted materials and macrophages are vital for successful cartilage regeneration 13. In our previous work, we found that HPCH can activate inflammatory responses and recruit macrophages. It is vital to modulate the activation states of macrophages to guide regeneration. It has been demonstrated that transforming growth factor-β (TGFβ) pathway is vital for the alternative macrophage (M2) activation 14. Hence, we posited that TGFβ1 could help to regulate the macrophage response induced by GM-HPCH.

MSCs are crucial cells for tissue regeneration 15. However, it is expensive to separate, culture and implant MSCs. MSC homing and migration towards lesion sites may be an alternative solution 16. Cumulative evidence shows that chitosan-based biomaterials activate macrophages to secrete bioactive molecules that stimulate MSC recruitment 17. It has also been demonstrated that TGFβ1 can promote chondrogenesis and recruit stem cells in the early injury stage 18. Collagen 19, gelatin 20, chitosan 21 and hyaluronic acid 22 incorporated with TGFβ1 have been developed for cartilage regeneration. Thus, TGFβ1 is a promising growth factor for inclusion in acellular hydrogels for cartilage regeneration 23, and it acts as a cell-homing-based hydrogel. However, most previous researches have focused on the chondrogenesis effect of the growth factor, showing only that TGFβ1 hydrogels can achieve better healing; little attention has been paid to the commutation among implanted materials, loaded growth factors and host immune response. Therefore, it is necessary to explore the underlying mechanism.

In this study, we successfully synthesized a novel thermosensitive and photocrosslinkable GM-HPCH hydrogel. TGFβ1 was loaded into the hydrogel to recruit MSCs, regulate macrophage activation and promote cartilage regeneration. The mild gelation conditions are essential for the bioactivity of the loaded growth factors. We further characterized the anti-apoptosis activity of GM-HPCH. The MSC-recruiting ability of GM-HPCH + TGFβ1 was investigated both *in vitro* and *in vivo*. Moreover, the composite hydrogel solution was injected into *in situ* cartilage defects, and 365 nm UV light was used to further crosslink the GM-HPCH. Cartilage regeneration in the defect was evaluated at 6 and 12 weeks postsurgery. We propose that this novel hydrogel system possesses advantages for cartilage tissue engineering.

## Methods

### GM-HPCH hydrogel preparation

**Figure 1:**
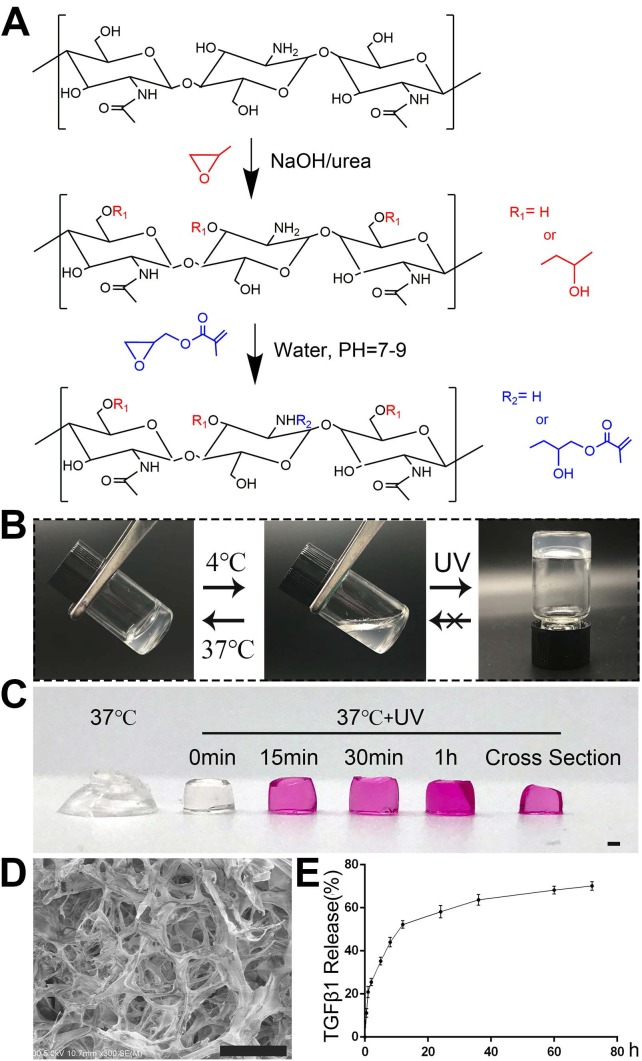
** Characterization of hydrogel morphology and properties.** (A) Synthesis process of GM-HPCH. (B) Sol-gel transition of GM-HPCH hydrogel. (C) Nutrient infiltration of GM-HPCH. Scale bar, 1 mm. (D) SEM characterization of GM-HPCH hydrogel after UV irradiation. Scale bar, 100 μm. (E) *In vitro* TGFβ1 release from the hydrogel by ELISA test.

The synthesis process of GM-HPCH was described in Figure 1A according to our previous work 11. Briefly, glycidyl methacrylate was added into the HPCH solution with stirring for 48 hours at pH 7-9. The solution was dialyzed and lyophilized to get the GM-HPCH. The TGFβ1 (Abcam, USA) was loaded into GM-HPCH under 4 ℃ via thoroughly stirring. Considering the slow release of TGFβ1 from the hydrogel and the optimal chondrogenesis, the loaded concentration of TGFβ1 was 1μg/mL 24.

### Scanning electron microscopy(SEM)

Pore morphology of the porous GM-HPCH was investigated by SEM (Quanta 250, FEI, USA). Samples were frozen by liquid nitrogen and snapped for cross-sectional imaging. After dehydrated via low-temperature drying, the samples were coated in gold performed with an accelerating voltage of 20 kV.

### *In vitro* TGFβ1 release

To quantitatively define the TGFβ1 release property of this hydrogel system *in vitro*, the TGFβ1-loaded GM-HPCH hydrogel was photocrosslinked for one minute and then was immersed into 1 mL PBS and incubated at 37 °C with a shaking rate of 100 rpm (n = 3). The time intervals were pre-determined at 0.0, 0.5, 1.0, 2.0, 5.0, 8.0, 12.0, 24.0, 36.0, 60.0, 72.0 h after incubation. The hydrogel suspension was collected and centrifuged at each predetermined time point. The fresh PBS was added into the tube for further drug release test. The concentration of TGFβ1 in the supernatant was tested using ELISA kit (Boster, China).

### Cell culture

Human umbilical vein endothelial cells (HUVECs), RAW264.7 and ATDC5 cells were purchased from American Type Culture Collection (ATCC), and cultured in DMEM/f12 (Hyclone, USA) supplemented with 10% fetal bovine serum (FBS), penicillin (100 U/ml) and streptomycin (100 mM). MSCs were extracted from 4 weeks SD rats and chondrocytes were extracted from neonatal rats according to previous reported 25, 26. MSCs and chondrocytes were cultured in DMEM/f12 supplemented with 10% FBS, penicillin (100 U/ml) and streptomycin (100 mM). All cells were cultured at 37 ℃ under a humidified atmosphere of 5% CO_2_. Passages 3 of MSCs and chondrocytes were used for the following experiments.

### Cell viability

The cell viability of MSCs cells encapsulated in the hydrogels after 3 days' culture was evaluated by a Live/Dead assay (Invitrogen) using confocal laser scanning microscopy (CLSM, Olympus FV 1000, USA). The cytotoxicity of GM-HPCH was evaluated in MSCs by CCK-8 assay according to manufacturer instruction. Briefly, cells were seeded in 96-well plates with a density of 5000 cells/well. 100 µL culture medium with 3 mg/ml GM-HPCH with 0.05% or 0.1% Irgacure 2959 (IC 2959) (Sigma-Aldrich, USA) was added, and the culture medium without hydrogel was used as a control. The medium changed every day. After incubation for 24, 48 and 96 hours, the medium was replaced by 10% CCK-8 solution (Boster, China) and incubated 2 hours at 37 °C. After that, the optical density (OD) was measured by a microplate reader (Thermo, USA) at 450 nm.

### Cell adhesion

GM-HPCH hydrogels (60 µL, 2% w/v) were formed in 96-well plates by photocrosslinking. MSCs, RAW264.7, HUVECs or chondrocytes were respectively cultured on the surfaces of GM-HPCH or plates (as control) at a density of 5000 cells/ well for one day. The cells were stained by Live/Dead assay to visualize cellular morphology and viability on the surface of hydrogel, and imaged by a fluorescence microscope (Evos FL Auto, USA).

### Anti-apoptosis of GM-HPCH

Apoptotic cells were analyzed by flow cytometry using the apoptosis kit (BD Biosciences, USA). Sodium nitroprusside (SNP), a donor of exogenous NO free radical 27, is a commonly used apoptotic stimulus for investigating injury-related osteoarthritis 28. To measure anti-apoptosis of GM-HPCH, chondrocytes were stimulated with 1 mM SNP, and treated with HPCH or GM-HPCH simultaneously for three hours. Cells were harvested and stained by FITC-Annexin V and PI and analyzed by measuring fluorescent signal for Alexa 488 and PI with flow cytometer (BD flow cytometer, USA). At least 7000 events were used to analyze. Also, the total RNA of RAW264.7 was collected and qPCR was used to analyze the apoptosis-related gene expression, including BCL2 associated X protein (Bax), cysteinyl aspartate specific proteinase3 (Caspase 3) according to the manufacture instruction. Briefly, the mRNA was converted to complementary DNA (cDNA) by ReverTra Ace® qPCR RT Kit (Toyobo, Japan) and the RT-PCR was carried out using Real-Time PCR Systems (Bio-Rad, USA). GAPDH was used to normalize the relative amount of gene transcripts. And all primers used in this work are listed in Table S1.

### Immunomodulation of GM-HPCH + TGFβ1 hydrogel

To detect the influence of the TGFβ1 loaded hydrogel on different states of macrophages, we stimulated RAW264.7 cells with lipopolysaccharide (LPS) (100 ng/mL) to induce M1-type macrophages, and the control group (M0) was cultured under normal conditions. TGFβ1 (10 ng/mL), GM-HPCH (3 mg/mL) and GM-HPCH + TGFβ1 (3 mg/mL) hydrogels were added to different types of macrophages for one day, and total RNA was collected and qPCR was used to analyze the expression of M1 (TNF-α, IL-1, iNOS, IL-6, CD86) and M2 (IL-10, Arg-1, CCL22, CD163, TGFβ1) related genes. The primers are also listed in Table S1. The immunomodulation of GM-HPCH and TGFβ1 was also evaluated *in vivo* through the histochemical sections. The surgery and implantation of hydrogel was the same as the test of MSC migration *in vivo*. CD163 was used as the marker of M2 macrophages. The CD163 positive cells were calculated (five images in one group).

### *In vitro* MSCs migration

To imitate the inflammatory environment in the repair process, RAW264.7 cells were cultured with TGFβ1 or GM-HPCH under the stimulation of LPS (100 ng/mL) for 6 hours. Extract medium from LPS stimulated RAW264.7 (PBS addition) was used as the control. The supernatant was collected and filtrated for the migration test. The total RNA of RAW264.7 were collected and qPCR was used to analyze the gene expression of MSCs migration-related genes. That is, extracellular high mobility group box 1 (HMGB1), monocyte chemotactic protein-1 (MCP-1 or CCL2), macrophage inflammatory protein-1 alpha (MIP-1α or CCL3), macrophage migration inhibitory factor (MIF), prostaglandin E synthase (PTGES) 29. The primers are listed in Table S1. MSCs migration was evaluated by the Transwell plate according to the protocol 30. Briefly, 5 × 10^4^ BMSCs were seeded on the upper chambers of 24-well plates (8 μm; Corning, USA), and the extract medium was in the lower chambers. After culturing for 24 hours at 37 °C, the migrated cells of the upper chamber were fixed with 4% paraformaldehyde. After staining with 0.5% crystal violet dye, the cells on the upper surface of the upper chamber were removed with a cotton swab. The migrated cells migrating on the lower surface were imaged and counted (five images per group).

### Chondrogenesis of GM-HPCH + TGFβ1 hydrogel

MSCs were cultured in or on the surface of GM-HPCH hydrogel. The DMEM supplement with 10% FBS, 1% penicillin/streptomycin, 1% ITS ×100 (Insulin- Transferrin-Sodium selenite, Sigma-Aldrich, USA), 50 μM ascorbic acid (Sigma-Aldrich, USA), 100 nM dexamethasone (Sigma-Aldrich, USA) and 10 ng/ml TGF β1 to induce the chondral differentiation. After 7 days of culturing, the total RNA was collected, and qPCR was used to analyze the gene expression of Collagen II (COL II), SRY (sex determining region Y)-box 9 (Sox9), Aggrecan (Acan) and Collagen I (COL I). The primers are listed in Table S1.

Chondrocytes were cultured in or on the surface of GM-HPCH hydrogel for 3 days and 7 days to evaluate the effect on chondrogenic differentiation. The supernatants were collected and GM-HPCH hydrogel containing chondrocytes was digested in 500μl of papain solution. The sulfated glycosaminoglycan (sGAG) content was measured by using 1,9-dimethylmethylene blue (DMMB; Sigma-Aldrich, USA) dye-binding assay and DNA content of the papain-digested samples was quantified using Hoechst Bisbenzimide 33258 dye assay (DNA Quantitation Kit, Fluorescence Assay, Sigma-Aldrich, USA) as previously described 31. The release of COL II was measured by an enzyme linked immunosorbent assay (ELISA) kit (Meimian, China) according to the manufacturer's instructions.

To evaluate the role of macrophages in TGFβ1-mediated chondrogenesis, the extract one-day culture medium from RAW264.7 cells, TGFβ1 (10 ng/mL)-treated cells, TGFβ1 + RAW264.7 cells, M1 macrophages (RAW264.7 induced by 100 ng/mL LPS) or M2 macrophages (RAW264.7 induced by 100 ng/mL IL-4) was collected. ATDC5, a chondrogenic cell line, and rat chondrocytes were used to evaluate the chondral effect. The cells were cultured with the extract medium for two days. The total RNA was collected and analyzed as we mentioned above and the primers were also listed in Table S1. Safranin O staining of the conditioned cultured chondrocytes was performed according to the manufactory instruction.

### *In vivo* experiment to verify GM-HPCH + TGFβ1 recruitment of MSCs in cartilage defect region

To observe whether GM-HPCH + TGFβ1 hydrogel could recruit BMSCs, the 3rd generation of BMSCs were co-incubated with green fluorescent protein (GFP) lentiviral expression vector (Genechem, China) for 24 hours 25. The GFP-labeled BMSCs were expanded until GFP expression was stable and there was minimal cell death. All animal experimental procedures were approved by the Animal Care and Use Committee, Huazhong University of Science and Technology, and the experimental procedures obeyed the relevant laws and ethical principles. Healthy male SD rats, aged approximately 3 months and weighing approximately 300 g, were used in this study. Before the operation, the animals were anesthetized with 1% pentobarbital sodium by intraperitoneal injection. Using a medial parapatellar incision, left knee joints were exposed. The cartilage defects at the femoropatellar groove were uniformly created using an electric drill, with a diameter of 2 mm and a depth of 2 mm. Hydrogel was smoothly injected into defects. After thermal gelation, hydrogel was photocrosslinked by the UV light (365 nm, 6 mW/cm^2^) for one minute. The patella was restored, and knee cavity was sutured. The surgical procedure was showed in Schema 1. The rats were randomly divided into 3 groups (5 rats/group): 1) Group C (defect control group), without implanting any materials and directly closed the articular cavity; 2) Group H (GM-HPCH scaffold alone); 3) Group T (1 μg/mL TGFβ1 + GM-HPCH). The rats were injected with GFP-labeled MSCs immediately after the knee cavity closure and were sacrificed after 3 days or 7 days. Knee samples was harvested and fixed in 4% paraformaldehyde for three days and decalcified in 10% ethylenediaminetetraacetic acid (EDTA) solution for 1 month.

To analyze the migration of fluorescent MSCs, the decalcified samples were put in liquid nitrogen to fast froze, then embedded in optimum cutting temperature (OCT) medium and balanced at -20 ℃ for 2 hours. The samples were sectioned using a slicing machine (Leica CM1950, Germany), and the section thickness was 10 μm. The nuclei were stained with DAPI and observed and photographed by fluorescence microscopy (Evos FL Auto microscope, USA).

### Establishment of the model of cartilage defect and repair with the GM-HPCH + TGFβ1 hydrogel

The osteochondral defect model was created as mentioned above, and SD rats were sacrificed after 6 weeks or 12 weeks (six rats in each group). The operated knees were harvested and photographed. International Cartilage Repair Society (ICRS) scoring system was used to score the defect site 32 (Table S2).

After decalcification, the specimens of cartilage repairing were dehydrated in a series of graded ethanol and embedded in paraffin. Decalcified specimens were then sectioned into 4 μm slices using a microtome (Leica, SM2000R). Safranin-O/Fast Green staining was used for the proteoglycan content and bone analysis. The sections were scored using a modified O'Driscoll histology scoring system (MODHS), which is a histological system for rating cartilage repair 33 (Table S3). COL I histochemistry staining were also analyzed. Digital images of the stained sections were obtained using the Evos FL Auto microscope.

### Statistical Analysis

All data are presented as mean ± standard deviation. All experiments were performed in at least three replicates. Statistical analysis was performed by using student's t-test or one-way ANOVA test to evaluate differences in different groups. The level of significance was set at P < 0.05. The data were analyzed using GraphPad Prism 6.0 (GraphPad Software, San Diego, CA, USA).

## Results and Discussion

Previous studies have demonstrated that growth-factor-loaded hydrogels can effectively repair chondral defects 34. However, little research has addressed how the loaded growth factors and implanted hydrogel influence the local reparation environment and cell migration and differentiation. In this study, a novel thermosensitive and photocrosslinkable hydrogel with good biocompatibility and biodegradability was used for cartilage tissue engineering. The shape adaption and stress resistance make it suitable for the irregular cartilage defect. Our works focus on host cell response of the implantation of GM-HPCH and the regulation of loaded drugs. We demonstrated that TGFβ1-loaded GM-HPCH could promote cartilage repair by exerting immunomodulatory effects, activating macrophages towards an M2 phenotype, anti-apoptosis, promoting MSC migration and providing a 3D growth environment for cell differentiation and secretion. Furthermore, we also confirmed the therapeutic effects of TGFβ1-loaded GM-HPCH in a rat model of chondral defects. The schematic repair process is shown in Schema 2. Overall, our data suggest that TGFβ1-loaded GM-HPCH has excellent potential as a novel biomaterial for cartilage regeneration.

### Characterization of thermosensitive photocrosslinkable GM-HPCH

The GM-HPCH synthesis process is illustrated in Figure 1A. The conventional vial-tilting method showed that our GM-HPCH solution transited to a gel phase at 37 °C in less than 1 min. This process can be reversed by resetting the temperature to 4 °C. The 365 nm UV treatment can further improve the mechanical properties 11, and maintain the shape of the hydrogel (Figure 1C). However, the gelation phase cannot be reversed even if the hydrogel is cooled down again (Figure 1B). The smart thermosensitive and photocrosslinkable product was applied as an injectable biomaterial, and it has advantages including fast *in situ* gelation to maintain the shape, and enhanced mechanical properties to resist pressure.

The GM-HPCH still possessed hierarchical pores after the UV treatment. These pores contain interconnective macropores with canals and micropores on their walls (Figure 1D). Due to the massive molecular weight of GM-HPCH, the mass fraction of the hydrogel solution is 2 wt%, which means that most of the hydrogel is water. The properties of the pores and the low mass fraction of the hydrogel provided desirable microenvironments for chondrocytes or BMSCs to survive. The inner channels provide an advantage for the communication of nutrients and cellular metabolic waste 35. The culture medium infiltration test showed that nutrients could rapidly permeate into the hydrogel (Figure 1C).

An ELISA kit was used to verify the release of TGFβ1. Following burst release over the first 2 hours, TGFβ1 was released smoothly due to the primary protection afforded by incorporation of the hydrogel (Figure 1E). When the TGFβ1-loaded hydrogel was implanted, the burst release provided high concentrations of the stem cell recruitment signal 18. The sustained release at low concentrations is necessary for the stem cells to differentiate towards chondrocytes and modulate macrophages. This release mode satisfied the cartilage regeneration process, which indicates that the hydrogel is a promising candidate for cartilage tissue engineering.

### Cell compatibility and adhesion on the hydrogel surface

**Figure 2:**
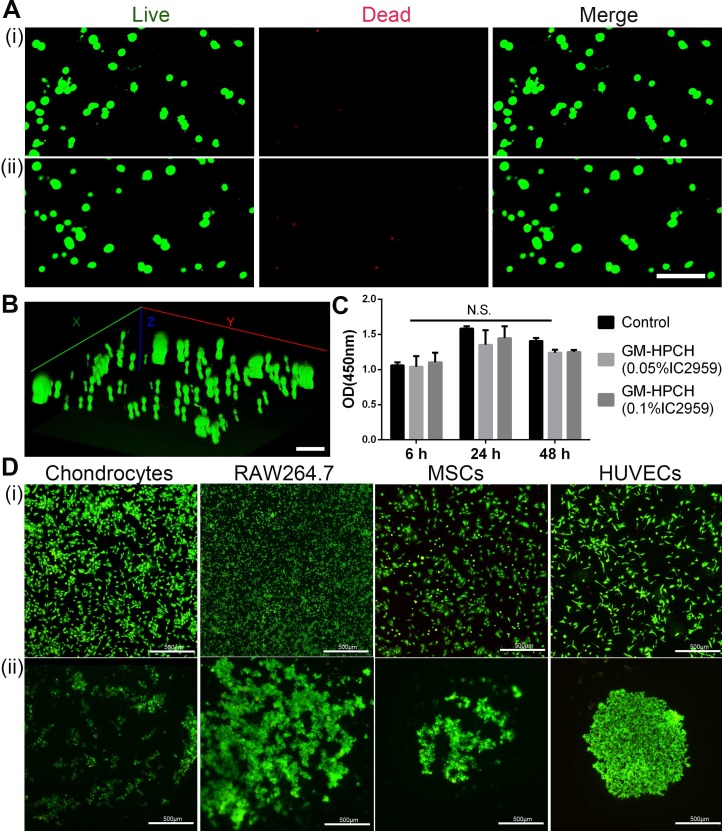
** Cell viability and adhesion of GM-HPCH.** (A) Live (green) /Dead (red) cells staining of MSCs cultured within GM-HPCH hydrogel over 3 days before (i) and after UV treatment (ii). Scale bars, 100 µm. (B) Three-dimensional reconstruction of the Live/Dead assay of MSCs after UV treatment. Scale bars, 100 µm. (C) CCK8 assay of extract culture medium with 3mg/ml GM-HPCH and 0.05% or 0.1% IC2959. N.S. indicates not significant in different groups. (D) The merge images of the Live/Dead assay of different cells adhered on the TCPS plate (i) or on GM-HPCH hydrogel (ii). Scale bars, 500 µm.

Cell viability is an essential property for biomaterial implantation. Factors that influence cell viability include the toxicity of materials and the nutrient exchange rate. Porosity plays an essential role in cell survival. As mentioned above, the interconnective pores benefit the nutrient communication. The toxicity of the photocrosslinking agent and the oxygen radicals released during the crosslinking process were the main problem for the application of photosensitive hydrogels 36. IC2959 is commonly used in the photocrosslinking process, and the applied concentration is usually in the range of 0.01~0.1% 37. Some researchers have proposed that phototoxicity can be reduced by lowering the concentration of the photocrosslinking agent, shortening the UV exposure time, and using a more reactive target 38. The results of the Live/Dead assay showed that there were few dead cells before and after the short UV treatment (1 min) (Figure 2A-B). We also used the CCK8 assay to confirm that there were no significant differences in cell viability between the treatments with 0.05% and 0.10% IC2959 (Figure 2C).

The three-dimensional (3D) hydrogels favoring cell survival and proliferation were potentially suitable biomimetic extracellular matrices (ECMs) for tissue regeneration. According to our previous research on cell adhesion to hydrogel surfaces 39, we found that HeLa cells, among other cell types, tend to aggregate on the surface. In this case, cell-cell communication was improved, which would favor cell function understimulation 40. Thus, we tested cell adhesion on GM-HPCH using different cell sources (Figure 2D). The results showed that all types of cells tended to form cell colonies on the surface. The aggregated state of chondrocytes and MSCs can improve cell-cell communication, which is vital for stem cell differentiation and chondrogenesis. RAW264.7 cells maintained their round shape and formed an aggregated colony, which indicated that GM-HPCH did not stimulate inflammation 41. In addition, HUVECs aggregated tightly and did not spread, indicating that the hydrogel did not favor endothelial cell adhesion to the hydrogel surface, which could prevent vascularization of the regenerated cartilage 42.

**Figure 3:**
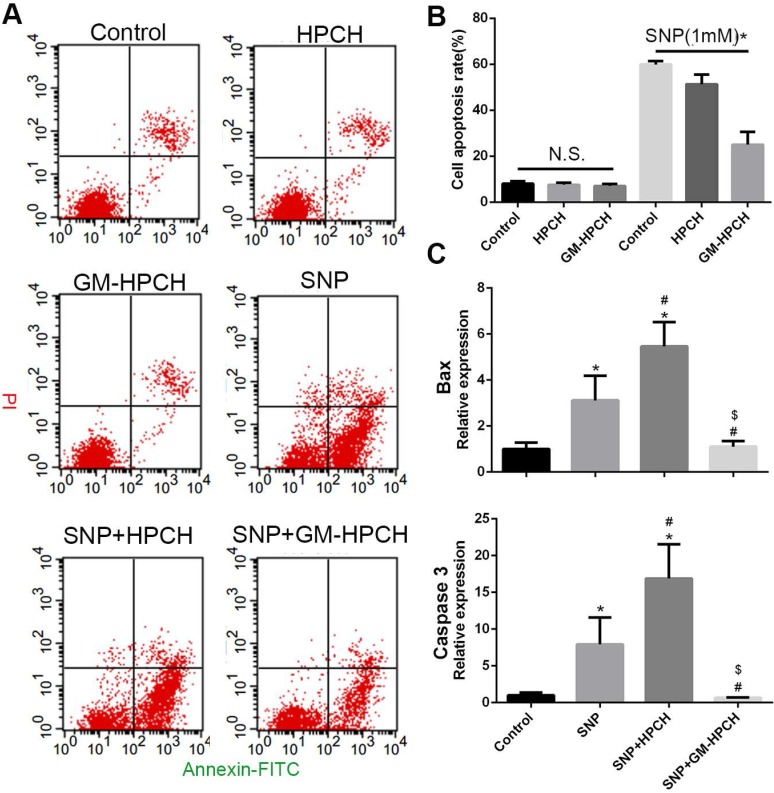
** Anti-apoptotic effects of GM-HPCH on chondrocytes in an inflammatory environment.** (A) Effect of GM-HPCH on the elevated rate of chondrocyte apoptosis induced by SNP. Representative scatter plots showed apoptosis levels determined by flow cytometry. (B) Apoptosis rate of GM-HPCH-treated apoptotic chondrocytes for three hours. *P < 0.05. (C) The expression of apoptosis-related genes (Bax and Caspase3) in GM-HPCH-treated apoptotic chondrocytes. *P < 0.05 versus control; #P < 0.05 versus SNP; $P < 0.05 versus SNP + HPCH.

The toxicity and anti-apoptosis activity of the GM-HPCH material was also evaluated using an apoptosis detection assay, and we found no significant difference in apoptosis rate among the HPCH and GM-HPCH on chondrocytes in normal conditions. Under the SNP-induced condition, GM-HPCH was found to inhibit chondrocytes from apoptosis, but HPCH did not have the same effect on chondral apoptosis (Figure 3A-B). The mRNA results of apoptosis-related genes (Bax and Caspase 3) showed that GM-HPCH could significantly suppress the expression of these genes (Figure 3C). The glycidyl methacrylate functional groups may account for the anti-apoptosis activity of the GM-HPCH, as the chemical crosslinking of GM-HPCH is radical-initiated chain polymerization, and free radicals generated from SNP can react with functional groups.

### GM-HPCH degradation *in vivo*

Hydrolytic and enzymatic *in vitro* degradation tests of the GM-HPCH hydrogel were performed in our previous work 11, but *in vivo* degradation tests had not yet been conducted. The degradation rate of hydrogels *in vivo* is vital for the regeneration of cartilage 43. To evaluate the degradation of GM-HPCH *in vivo*, a subcutaneous Sprague-Dawley (SD) rat model was used and tested after 1, 3, and 6 weeks. Scant hydrogel was degraded after 1 week. Inflammatory cells aggregated around and infiltrated into the hydrogel. After 3 weeks, most of the hydrogel was degraded, and inflammatory cells remained around the hydrogel. At 6 weeks, the hydrogel was fully degraded, and no inflammatory cells were detected. More importantly, no fibrous tissue was observed at the implanted site, which indicates that the hydrogel can be entirely removed (Figure S1). The role of GM-HPCH is a temporally matrix to promote cartilage regeneration. The migrated host cells and the regulation of local environment were supposed to participate the cartilage regeneration process after degradation of the hydrogel. Other studies have shown that byproducts during degradation are beneficial to cartilage repair 44. Thus, it is reasonable to use GM-HPCH as an implanted hydrogel for *in situ* cartilage regeneration.

### Immunomodulation of GM-HPCH + TGFβ1 hydrogel

**Figure 4:**
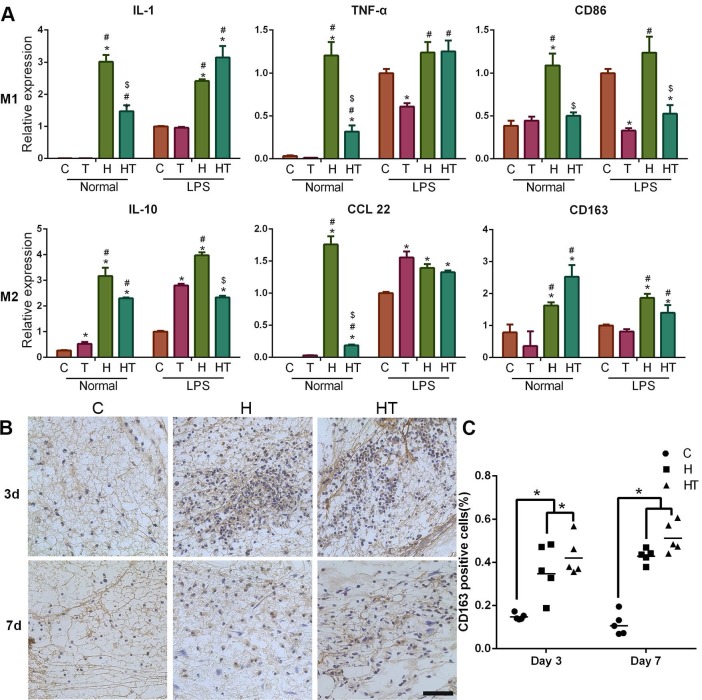
** Immunomodulation of the GM-HPCH and TGFβ1 hydrogel.** (A) The relative mRNA transcription of genes related to M1 (IL-1, TNF-α, CD86) and M2 (IL-10, CCL22, CD163) in RAW264.7 cells cultured with GM-HPCH and TGFβ1 for 24 hours. RAW264.7 cells were either prestimulated into M1 using LPS (100 ng/mL), or used as control without stimulation. Data are expressed as the mean ± SD. *P < 0.05 versus the control; #P < 0.05 versus TGFβ1; $P < 0.05 versus GM-HPCH. (B) Histological sections of the defect were stained for M2 macrophages using a CD163^+^ antibody. (C) Quantification of the ratio of CD163 positive cells in the defect. Five images were calculated in each group. *P < 0.05. Scale Bars: 50 μm. C, PBS control. T, TGFβ1. H, GM-HPCH. HT, GM-HPCH + TGFβ1.

A timely and smooth transition from the inflammatory stage to the healing stage is vital for tissue engineering. Smart biomaterial with precise control of the M1-to-M2 macrophage transition could ensure this process 45. Various studies have explored the controlled release strategies and physical/mechanical cues to tune this M1/M2 balance 46. In our experiments, we found that GM-HPCH can increase both the M1- and M2- related gene expression in normal conditions. TGFβ1 addition can modulate the inflammatory response of GM-HPCH. Specifically, qPCR results showed that GM-HPCH could improve both M1 (iNOS, IL-1, TNF-α, IL-6, CD86) and M2 (Arg-1, IL-10, CCL22, CD163) gene expression related to the presence of macrophages, as observed in the M0 (normal condition) states. Importantly, under the M1 state (LPS stimulation), the improvement of M1-related gene expression was not significant, whereas GM-HPCH can still significantly improve the expression of M2-related genes. The presence of TGFβ1 can significantly suppress the M1-related gene expression induced by the hydrogel under different states of macrophages, but not M2-related gene expression (Figure 4A, Figure S2A-C). Our immunohistochemical staining results also confirmed that higher amounts of CD163 positive cells aggregated in the GM-HPCH + TGFβ1 and GM-HPCH groups at 3 days and 7 days' postsurgery, which indicates successful activation of M2 macrophages (Figure 4B-C). These results indicate that the combination of GM-HPCH and TGFβ1 favored the M1-to-M2 transition. Interestingly, both GM-HPCH and TGFβ1 can improve the expression of TGFβ1 in macrophages (Figure S2D), which may benefit chondrogenesis.

### MSC homing of the GM-HPCH + TGFβ1 hydrogel

**Figure 5:**
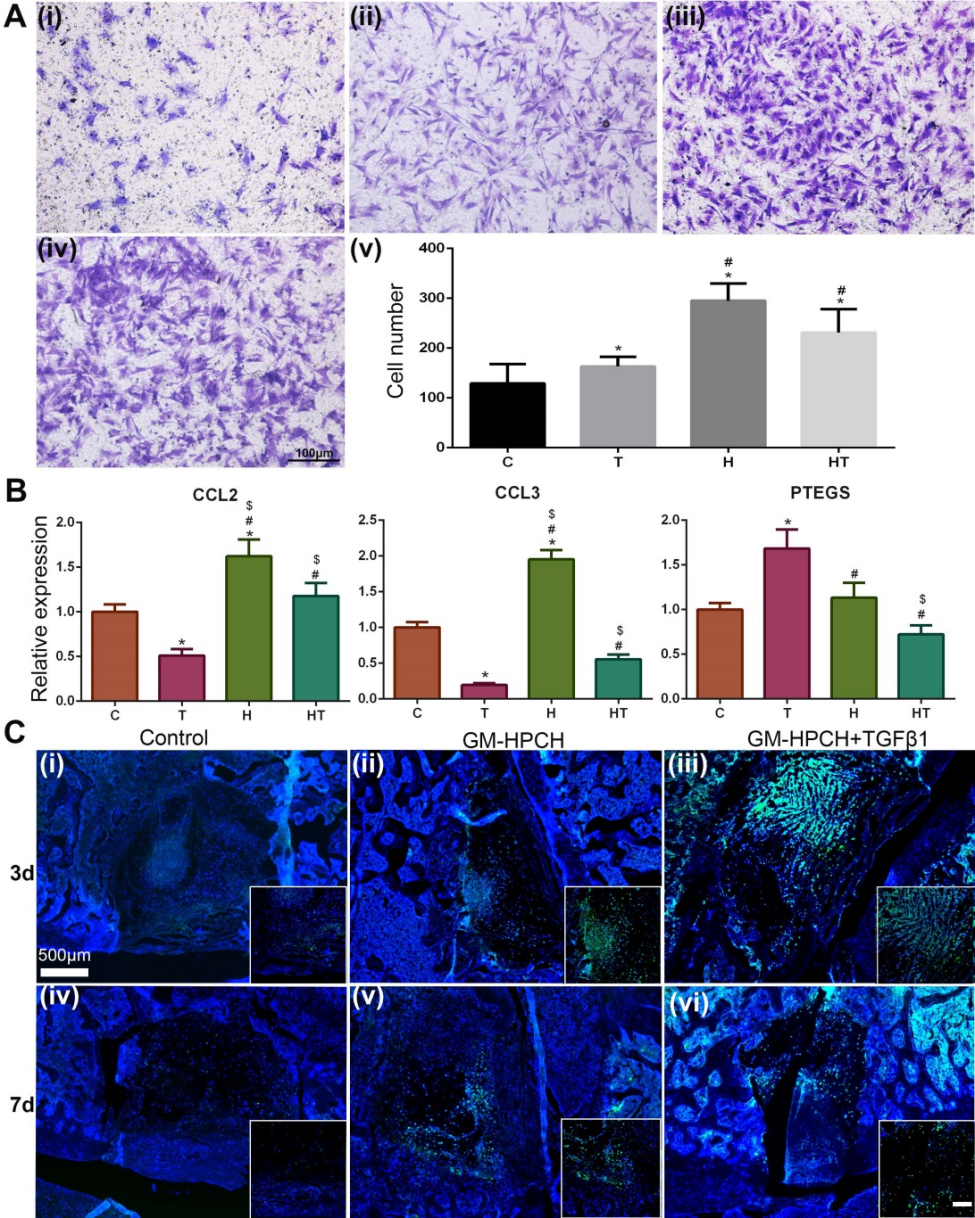
** MSC homing of GM-HPCH + TGFβ1.** (A) *In vitro* MSC migration test using Transwells, where (i) is the control (extracted culture medium from the LPS-stimulated RAW264.7 cells when treated with PBS), (ii-iv) is the extracted culture medium from the LPS-stimulated RAW264.7 cells when treated with TGF β1 or GM-HPCH or both, and (v) is the quantification of the migrated MSCs after 24 hours' culture. (B) The relative mRNA transcription of MSCs migration genes (CCL2, CCL3 and PTGES) in RAW264.7 cells treated with GM-HPCH and TGFβ1. (C) *In vivo* MSC migration test 3 days (i - iii) and 7 days (iv - vi) after implantation. The injected MSCs were labeled with GFP to monitor the migration. Scale bar in the enlarged box is 200 μm. *P < 0.05 versus the control; #P < 0.05 versus TGFβ1; $P < 0.05 versus GM-HPCH. C, PBS control. T, TGFβ1. H, GM-HPCH. HT, GM-HPCH + TGFβ1.

Although chondrocytes can barely proliferate due to the terminally differentiated state, it is controversial to encapsulate stem cells in hydrogel for tissue engineering applications 47. Stem cell encapsulation can achieve better tissue regeneration, but the time-consuming nature and high cost of this manipulation limits its applications. Furthermore, the viability of the transplanted cells is low because insufficient nutrition infiltrates into the hydrogel after implantation. Thus, acellular hydrogels draw great attention, and it is important to recruit stem cells from the host to achieve successful regeneration 48. For our acellular GM-HPCH hydrogel, *in vitro* MSC migration experiments showed that the conditioned medium of RAW264.7 cells stimulated by GM-HPCH and TGFβ1 could also promote MSC migration (Figure 5A). The total RNA was collected, and qPCR was used to analyze the gene expression associated with the MSC migration (CCL2, CCL3, HMGB1, MIF, PTGES). The results showed that GM-HPCH could significantly increase CCL2 and CCL3 expression, and TGFβ1 could increase PTGES expression (Figure 5B). No significant differences were observed between HMGB1 and MIF expression levels (Figure S3). These results indicate that GM-HPCH and TGFβ1 could stimulate macrophages to promote MSC migration through CCL2, CCL3 and PTGES activation.

To evaluate MSC homing of the GM-HPCH + TGFβ1 hydrogel *in vivo*, we implanted the hydrogel into the chondral defect; after the operation, GFP-labeled MSCs were injected into articular cavities. Both the 3- and 7-day results of the GM-HPCH and GM-HPCH+ TGFβ1 groups showed aggregation of fluorescent-labeled MSCs at the defect site (Figure 5C). The results show that GM-HPCH + TGFβ1 hydrogel can promote MSCs homing for cartilage regeneration.

### Chondrogenesis of GM-HPCH + TGFβ1 hydrogel

**Figure 6:**
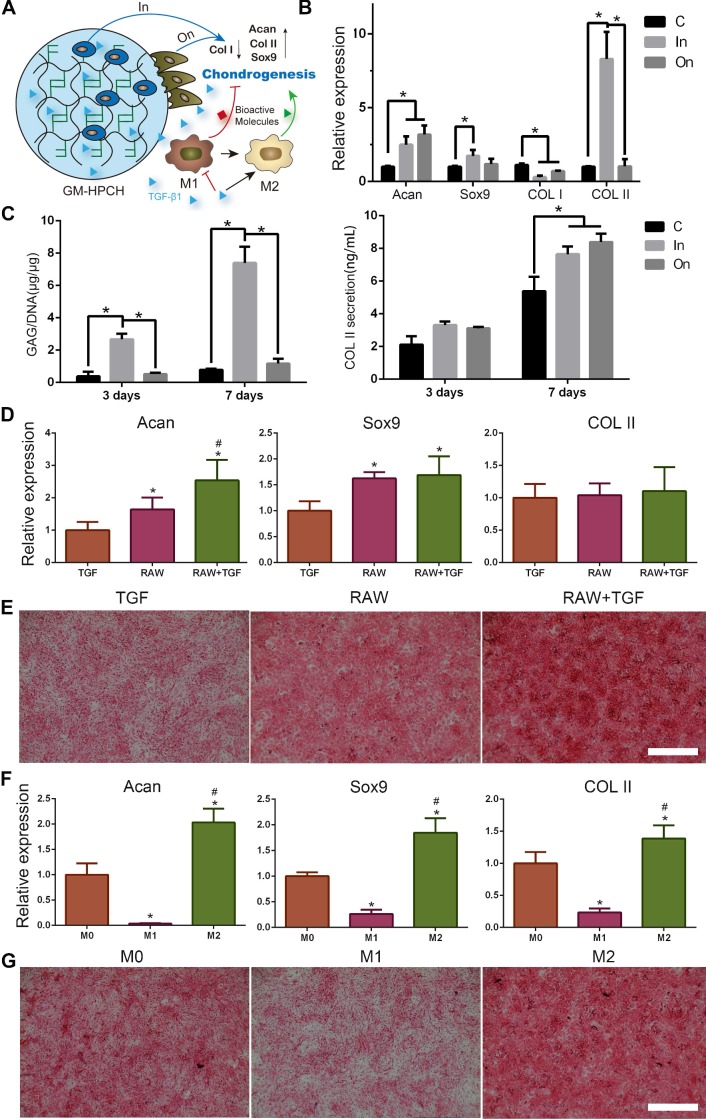
** Chondrogenesis of GM-HPCH + TGFβ1.** (A) Schematic illustration of the interaction between GM-HPCH and macrophages for chondrogenesis. (B) The relative mRNA transcription of chondrogenic genes (Acan, Sox9, COL II and COL I) when MSCs were cultured in or on the surface of GM-HPCH. *, P < 0.05. (C) GAG and COL II secreted by chondrocytes cultured in or on hydrogel at 3 days and 7 days. *P < 0.05. The relative mRNA transcription of chondrogenic genes (Acan, Sox9 and COL II) in chondrocytes (D) and Safranin O staining (E) when treated with the extract medium form RAW264.7 cells and TGF β1. *P < 0.05 versus TGFβ1; #P < 0.05 versus RAW264.7 cells. The relative mRNA transcription of chondrogenic genes (Acan, Sox9 and COL II) in chondrocytes (F) Safranin O staining (G) when treated with extract medium from M0 macrophages, M1 macrophages or M2 macrophages. *P < 0.05 versus M0; #P < 0.05 versus M1. Scale bar, 400 μm.

Improved cell-cell contact can be beneficial for stem cell differentiation 49, so we harvested the total RNA of MSCs that we cultured on GM-HPCH to test the cartilage related gene expression (Acan, Sox9, COL II and COL I). To examine whether the 3D culture environment can promote cartilage secretion 50, MSCs were then encapsulated in hydrogel for 7 days to test the same cartilage related genes. The qPCR results show that Acan, Sox9 and COL II expression increased in both groups compared with the control. Importantly, COL I expression decreased in both groups, especially when cultured in the hydrogel (Figure 6B), which indicates that the hydrogel can protect the cartilage from hypertrophy. These results indicated that GM-HPCH might promote hyaline cartilage formation, rather than fibrocartilage. To evaluate the effect on chondrogenic differentiation, we also cultured chondrocytes in or on the GM-HPCH and evaluated GAGs and COL II secretion. At 3 days, the chondrocytes encapsulated in GM-HPCH secreted more GAGs compared with other groups, and the same phenomenon was found after 7 days. For COL II secretion, there was no significant difference at 3 days, but the significant increase appeared at 7 days when chondrocytes were cultured in or on the hydrogel (Figure 6C). The results were consistent with the PCR, which indicated that GM-HPCH could promote the chondrogenic differentiation, especially when cells encapsulated in the hydrogel.

TGFβ1 is known to effectively induced chondrogenesis of MSCs 51 (Figure S4). Previous work has reported that macrophages are crucial in TGFβ1-mediated chondrogenesis 52. To verify whether the interaction of TGFβ1 with macrophages can promote chondrogenesis, culture medium extracted from TGFβ1 and macrophages was used to evaluate the effect on the chondrogenic differentiation of ATDC5 and chondral gene expression of chondrocytes. The RT-PCR results show that the RAW264.7 + TGFβ1 group could promote chondral gene expression of Acan and Sox9, compared with the RAW264.7 or TGFβ1 groups (Figure 6D) (Figure S5A). We also found that when macrophages were induced to the M1 type, the expression of chondral genes (Acan, Sox9 and COL II) was suppressed. However, when macrophages were induced to the M2 type, the promotion of chondral gene expression (Acan, Sox9 and COL II) was observed (Figure 6F) (Figure S5B). Safranin O staining of the conditioned cultured chondrocytes is consistent with the PCR results (Figure 6E, G). It is reasonable to propose that polarization of macrophages to M2 caused by TGFβ1 may be responsible for the promotion of chondrogenesis. Further work is needed to prove this relationship in the future.

TGFβ1 has been reported to be associated with osteoarthritis and osteophyte formation 53. It is necessary to restrict TGFβ1 to a local region instead of the whole knee cavity, because abnormal stimulation is responsible for osteophyte formation 54. In our work, TGFβ1 was loaded into GM-HPCH by slow release. When injected into the cartilage defect, the hydrogel gelled once the temperature changed and was photocrosslinked by UV light, ensuring the local application of TGFβ1. The slow release of TGFβ1 over several days could modulate the local immune response and initiate chondrogenesis.

### Chondral defect repairing of GM-HPCH + TGFβ1 hydrogel

**Figure 7:**
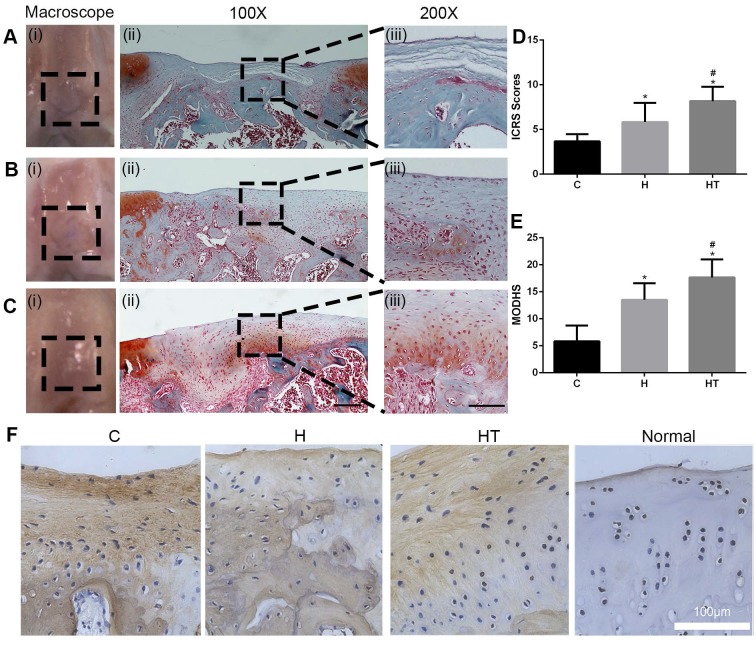
** Histological evaluation of *in vivo* cartilage regeneration of the GM-HPCH + TGFβ1 hydrogel in defects after 12 weeks.** (A) PBS control group, (B) GM-HPCH and (C) GM-HPCH + TGFβ1.The macroscopic view (i) and Safranin O-Fast green staining in 100 X (ii) and 200 X (iii) magnification are presented in each group. The scale bar is 400 μm in 100 X and 200 μm in 200 X. (D) ICRS visual histological evaluations of repaired cartilages. (E) MODHS histological evaluations of repaired cartilages. (F) Immunohistochemical staining of COL I of repaired cartilages in different groups after 12 weeks. Scale bar, 100 μm. Data are presented as the mean ± SD (n = 6); *P < 0.05 versus the control; #P < 0.05 versus GM-HPCH. C, PBS control. H, GM-HPCH. HT, GM-HPCH + TGFβ1.

The macroscopic evaluation at both 6 weeks and 12 weeks showed that the regenerated cartilage was smoother in the GM-HPCH and GM-HPCH + TGFβ1 groups than in the control group (Figure 7A-C(i)) (Figure S6). The International Cartilage Repair Society (ICRS) scores increased in the control, GM-HPCH and GM-HPCH + TGFβ1 groups, and there was a significant difference among them (Figure 7D). The Safranin-O results reveal that TGFβ1-loaded hydrogel could promote hyaline cartilage-like tissue regeneration, compared with the hydrogel without TGFβ1 and the control group (Figure 7A-C(ii-iii)) (Figure S6A-C). However, the reparative effect of the GM-HPCH group was also better than that of the empty control group, which indicates that the GM-HPCH hydrogel itself contributed to the regeneration of cartilage. Interestingly, although the cartilage regeneration of GM-HPCH was as good as that in the GM-HPCH + TGFβ1 group at 6 weeks (Figure S6B-C), the regenerated cartilage degraded at 12 weeks in the GM-HPCH group, whereas a high concentration of proteoglycans and orientation distribution were still present in the GM-HPCH + TGFβ1 group (Figure 7B-C). These results indicate that GM-HPCH can promote cartilage regeneration, and TGFβ1 may play an essential role in preventing degradation in the new cartilage, possibly through the immunomodulation of macrophages.

Subchondral bone change is regarded as an essential factor determining the progress of osteoarthritis 55, so reconstruction of intact subchondral bone is necessary to prevent regenerated cartilage from degenerating 56. In our work, both the GM-HPCH and TGFβ1-loaded GM-HPCH groups achieved intact subchondral bone, which indicates high-quality cartilage regeneration (Figure 7A-C(ii)) (Figure S6A-C). The ICRS visual and MODHS histological evaluations also showed superior reparative effects in the GM-HPCH and GM-HPCH + TGFβ1 groups (Figure 7E) (Figure S6D-E). The immunochemical results also indicated decreased COL I in the GM-HPCH and GM-HPCH + TGFβ1 groups at 6 weeks and 12 weeks (Figure 7F) (Figure S6F), consistent with the *in vitro* PCR results showing COL I gene expression (Figure 6B).

In our work, we developed an acellular cell-homing GM-HPCH + TGFβ1 hydrogel to activate the host stem cells. The hydrogel can react with the host cells, recruiting stem cells and macrophages to participate in the regeneration process. This approach is straightforward, ready-to-use and effective.

## Conclusions

We developed and characterized a GM-HPCH + TGFβ1 hydrogel for cartilage regeneration. The incorporation of TGFβ1 into the GM-HPCH hydrogel can prolong its release and enhance its therapeutic effects. The GM-HPCH + TGFβ1 hydrogel increased MSC homing and differentiation, contributing to cartilage repair after injury. The immunomodulation of the GM-HPCH + TGFβ1 hydrogel increased macrophage M2 polarization at the site of the injury, which was identified to promote cartilage regeneration. Furthermore, the spatial distribution and anti-apoptosis activity were also beneficial for chondrogenesis. In summary, these findings highlight the potential of GM-HPCH + TGFβ1 hydrogel as a novel therapeutic strategy for cartilage tissue engineering.

## Supplementary Material

Supplementary methods, figures, and tables.

## Figures and Tables

**Schema 1:**
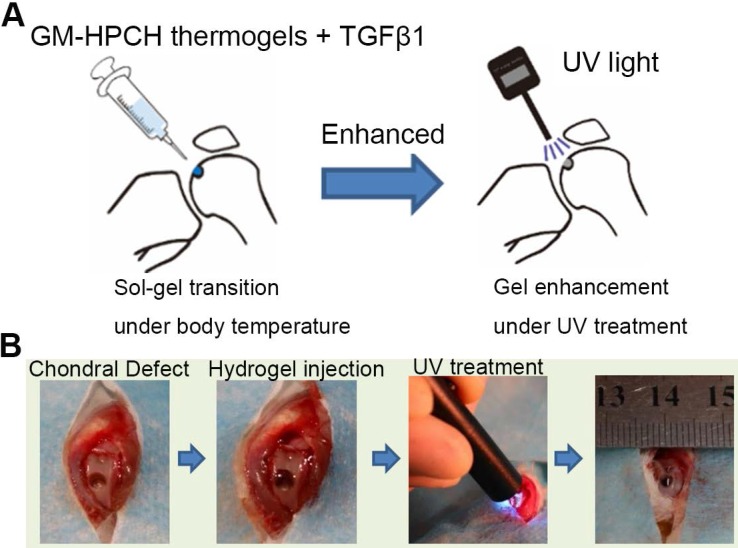
** The procedure of GM-HPCH hydrogel injection into the chondral defect.** (A) The schematic illustration. (B) The surgery process of GM-HPCH + TGFβ1 implantation.

**Schema 2:**
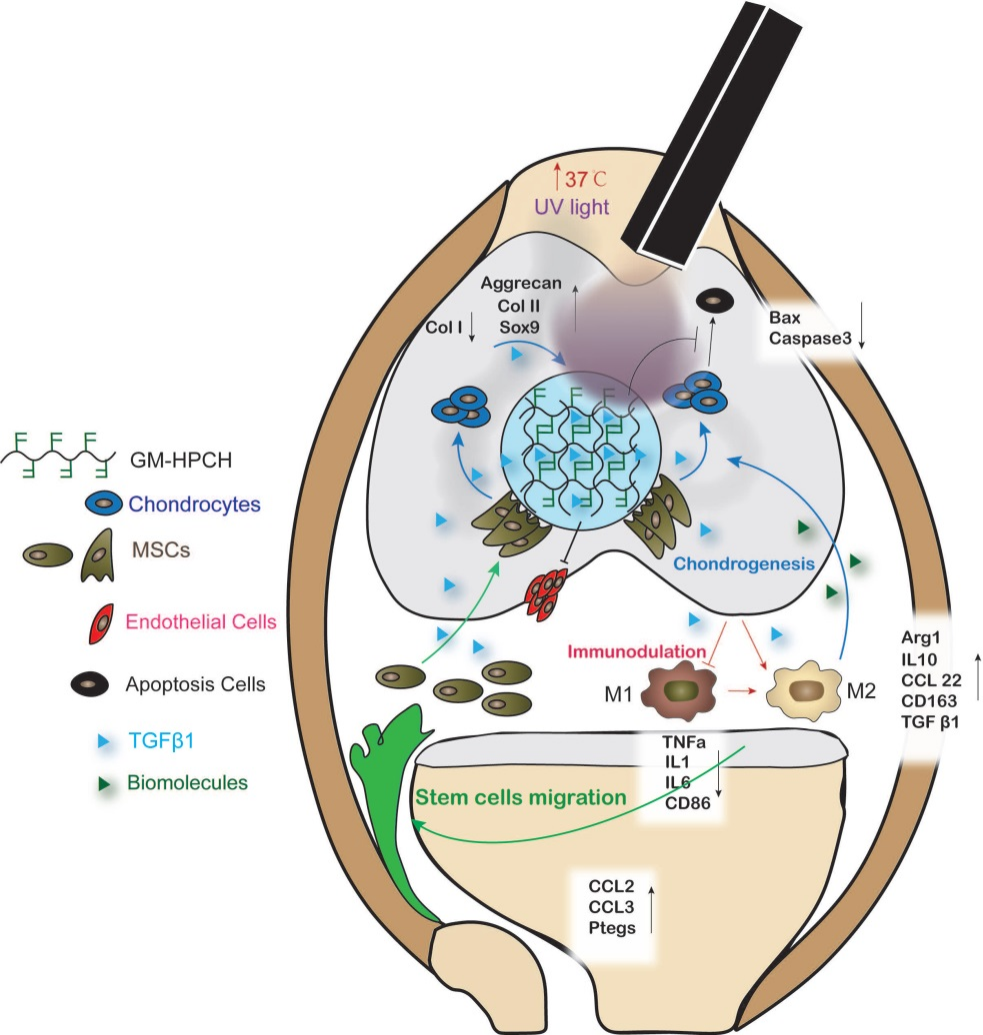
** Schematic graph demonstrating the cartilage repair mediated by GM-HPCH + TGFβ1 via immunomodulating activation of M2 macrophages, recruitment of stem cells and promotion of chondrogenesis.** First, the GM-HPCH injected on the chondral defects gels quickly when the temperature is increased, and the sequential application of UV light can enhance the mechanical properties of the hydrogel. The anti-apoptosis activity of the GM-HPCH hydrogel can prevent inflammatory-environment-induced chondrocyte apoptosis. HUVECs cannot adhere on the surface of the hydrogel, which indicates that the GM-HPCH can protect the regenerated cartilage from vascularization. Second, GM-HPCH + TGFβ1 acted as a cell homing site for cartilage. GM-HPCH can react with host macrophages to recruit MSCs, and TGFβ1 released from the hydrogel shifts the macrophages towards the M2 phenotype and facilitates chondrogenesis. Third, GM-HPCH + TGFβ1 established a microenvironment with the matrix secretion of native chondrocytes from adjacent cartilage and the release of TGFβ to favor cartilage regeneration.

## Abbreviations

Acan: aggrecan
Bax: BCL2 associated X protein
COL I: collagen I
IC2959: irgacure 2959
MSCs: marrow stromal cells
Sox9: (sex determining region Y)-box 9
ATCC: American Type Culture Collection
COL II: collagen II
cDNA: complementary DNA
CLSM: confocal laser scanning microscopy
Caspase 3: cysteinyl aspartate specific proteinase3
EDTA: ethylenediaminetetraacetic acid
HMGB1: extracellular high mobility group box 1
FBS: fetal bovine serum
GM-HPCH: glycidyl methacrylate-modified HPCH
GFP: green fluorescent protein
HUVECs: human umbilical vein endothelial cells
HPCH: hydroxypropyl chitin hydrogel
ICRS: international Cartilage Repair Society
LPS: lipopolysaccharide
MIP-1α: macrophage inflammatory protein-1 alpha
MIF: macrophage migration inhibitory factor
HAMA: methacrylated hyaluronic acid
MODHS: modified O'Driscoll histology scoring system
MCP-1: monocyte chemotactic protein-1
OD: optical density
PNIPAAm: Poly(N-isopropylacrylamide)
PTGES: prostaglandin E synthase
SEM: scanning electron microscopy
SNP: sodium nitroprusside
SD: Sprague-Dawley
TGFβ1: transforming growth factor-β1
