# Supplementary material for: Cartilage repair mediated by thermosensitive photocrosslinkable TGFβ1-loaded GM-HPCH via immunomodulating macrophages, recruiting MSCs and promoting chondrogenesis
Source: Theranostics. 2020 Feb 3;10(6):2872–87. doi: 10.7150/thno.41622 (PMC7052899; doi:10.7150/thno.41622)
Supplement: Supplementary file 1 — Supplementary methods, figures, and tables. [file thnov10p2872s1.pdf]

Supporting Information

Cartilage repair mediated by thermosensitive photocrosslinkable TGFβ1-loaded GM-HPCH via immunomodulating macrophages, recruiting MSCs and promoting chondrogenesis

Xiongfa Ji[1,3] , Zehua Lei[1*], Meng Yuan[2], Hao Zhu[1], Xi Yuan [1,], Wenbin Liu[1], Hongxu Pu[1], Jiawei Jiang[1], Yu Zhang[3✉], Xulin Jiang[2✉], Jun Xiao[1✉]*

1.Department of Orthopaedic Surgery, Tongji Hospital, Tongji Medical College, Huazhong University of Science and Technology, Wuhan, 430030, China

2.Key Laboratory of Biomedical Polymers of Ministry of Education & Department of Chemistry, Wuhan University, Wuhan, 430072, China.

3.Department of Orthopedics, Guangdong General Hospital, Guangdong Academy of Medical Sciences, Guangzhou, Guangdong 510080, PR China

*These authors contributed equally to this work.

✉ Jun Xiao, Xulin Jiang, and Yu Zhang are the co-corresponding authors.

Methods

Fluorescent labeling MSCs using GFP-lentivirus

GFP-lentivirus (Genechem, China) was used to label MSCs [1]. The 3rd passage MSCs were seeded into 96-well plates at 3×10^4 cells/mL. The cells were infected with GFP-lentivirus at a multiplicity of infection (MOI) of 1, 10, 100 for 12 h. The infectious efficiency was evaluated based on GFP expression after 72 h, observed using a fluorescent microscope. An MOI = 100 was selected for further experiments, because of sufficient GFP expression with minimum damage. Two more passage culture were performed to stable the GFP expression in MSCs for the following experiments.

Figure and Legend

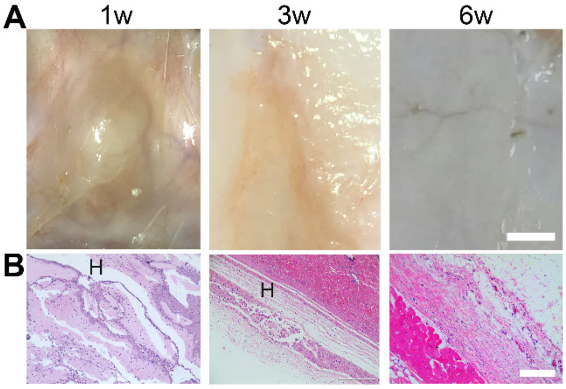

Figure S1 Degradation of GM-HPCH hydrogels (2 wt%) in vivo after 1 week, 3 weeks and 6 weeks. (A) the macroscopic view. Scale bar, 500 mm. (B) the H&E staining. Scale bar, 400 μm.

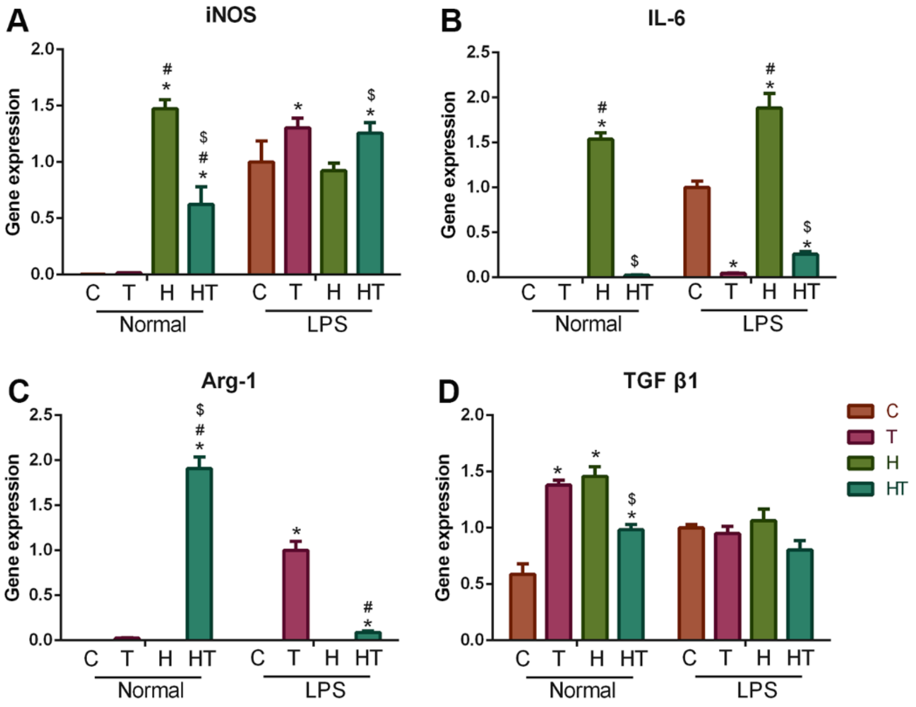

Figure S2 Immunomodulation of GM-HPCH + TGFβ1 hydrogel. (A) The relative mRNA transcription of M1 (iNOS, IL-6) and M2 (Arg-1, TGFβ1) related genes in RAW264.7 cultured with GM-HPCH and TGFβ1 for 24 hours. RAW264.7 was either pre-stimulated into M1 using LPS (10ng/mL), or used as control without stimulation. Data are expressed as mean ± SD. *$P < 0.05$ versus Control; #$P < 0.05$ versus TGFβ1; \$$P < 0.05$ versus GM-HPCH.

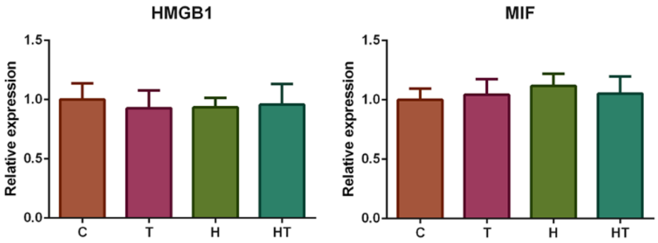

Figure S3 qPCR of cell-migration related genes. There was no significant difference in MIF and HMBG1 gene expression. C, PBS control. T, TGFβ1. H, GM-HPCH. HT, GM-HPCH+ TGFβ1.

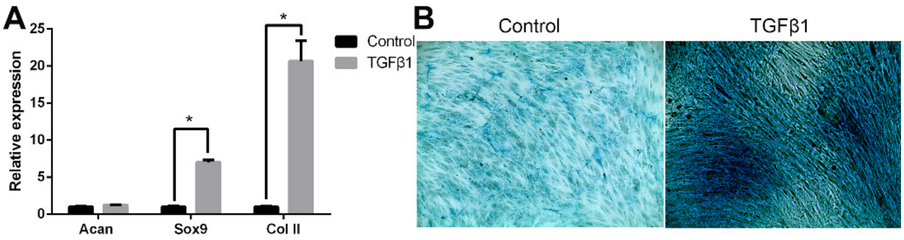

Figure S4 Chondrogenesis of TGF β1 for MSCs. (A) The relative mRNA transcription of chondrogenic genes (Acan, Sox9, COL II). (B) Alcian blue staining for MSCs after 14-day chondral induction. *$P < 0.05$.

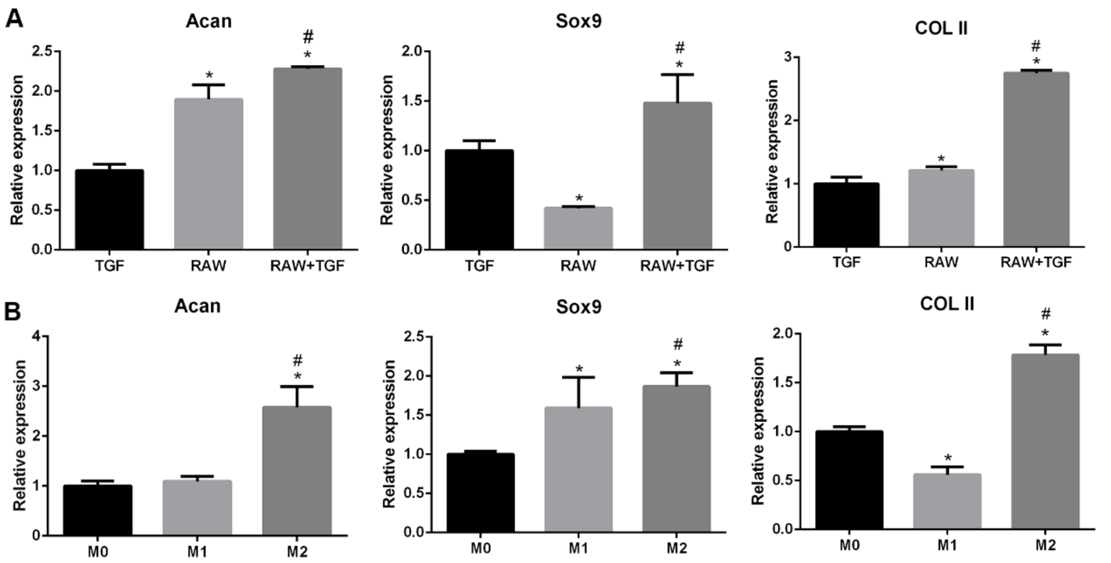

Figure S5 Chondrogenesis of GM-HPCH + TGFβ1 for ATDC5. (A) The relative mRNA transcription of chondrogenic genes (Acan, Sox9 and COL II) in ATDC5 treated with the extract medium form RAW264.7 and TGF β1. *P < 0.05 versus TGFβ1; #P < 0.05 versus RAW264.7. (B) The relative mRNA transcription of chondrogenic genes (Acan, Sox9 and COL II) in ATDC5 treated with extract medium from M0 macrophages, M1 macrophages or M2 macrophages. *P < 0.05 versus M0; #P < 0.05 versus M1.

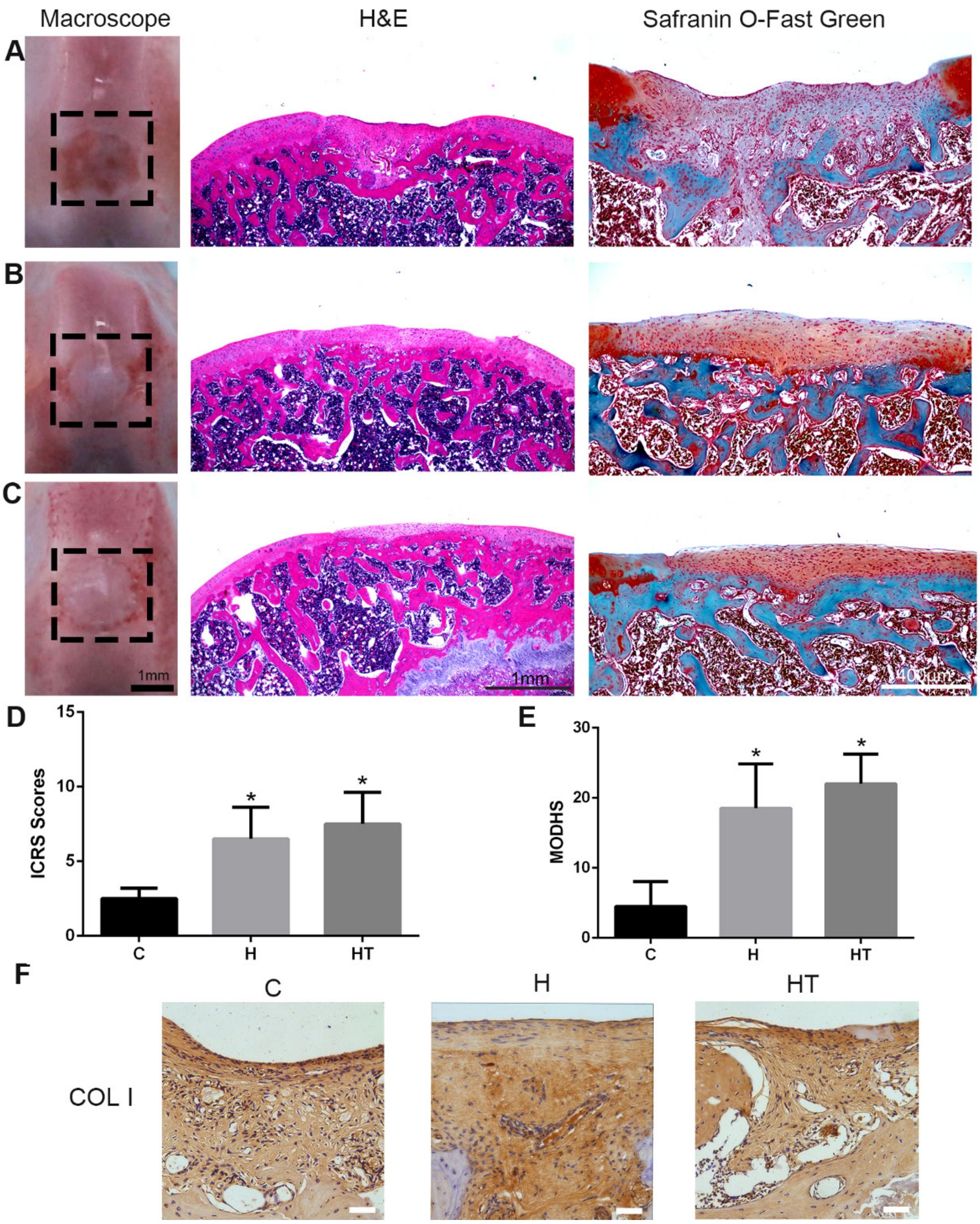

Figure S6 Histology evaluation of in vivo cartilage regeneration of GM-HPCH+ TGFβ1 hydrogel in defects after 6 weeks. (A) was PBS control group, (B) was GM-HPCH and (C) was GM-HPCH+ TGFβ1 group. The macroscopic view, H&E and SafraninO-Fast green staining were presented in each group. The scale bar was 1000 μm in H&E staining and 400 μm in Safranin O-Fast green staining. (D) ICRS visual histological evaluations of repaired cartilages. (E)MODHS histological evaluations of repaired cartilages. (F) Immunohistochemical staining of COLI of repaired cartilages in different groups after 6 weeks. Scale bar, 50 μm. Data are presented as mean

± SD (n = 3); *P < 0.05 versus control; #P < 0.05 versus GM-HPCH. C, PBS control. H, GM-HPCH. HT, GM-HPCH + TGFβ1.

Table S1 The primers sequences.

Gene	Species		Primers(5'-3')
Bax	Mouse	Forward	AGACAGGGGCCTTTTTGCTAC
		Reverse	AATTCGCCGGAGACACTCG
BCL 2	Mouse	Forward	GCTACCGTCGTGACTTCGC
		Reverse	CCCCACCGAACTCAAAGAAGG
Caspase 3	Mouse	Forward	CTCGCTCTGGTACGGATGTG
		Reverse	TCCCATAAATGACCCCTTCATCA
IL1β	Mouse	Forward	CCCAACTGGTACATCAGCACCTC
		Reverse	GACACGGATTCCATGGTGAAGTC
TNF-α	Mouse	Forward	GGACTAGCCAGGAGGGAGAA
		Reverse	CGCGGATCATGCTTTCTGTG
IL6	Mouse	Forward	CTGCAAGAGACTTCCATCCAG
		Reverse	AGTGGTATAGACAGGTCTGTTGG
CD86	Mouse	Forward	TCAATGGGACTGCATATCTGCC
		Reverse	GCCAAAATACTACCAGCTCACT
Arg-1	Mouse	Forward	CTCCAAGCCAAAGTCCTTAGAG
		Reverse	GGAGCTGTCATTAGGGACATCA
IL10	Mouse	Forward	GCTCTTACTGACTGGCATGAG
		Reverse	CGCAGCTCTAGGAGCATGTG
CD163	Mouse	Forward	ATGGGTGGACACAGAATGGTT
		Reverse	CAGGAGCGTTAGTGACAGCAG
CCL22	Mouse	Forward	CTCTGCCATCACGTTTAGTGAA
		Reverse	GACGGTTATCAAAACAACGCC
TGF β1	Mouse	Forward	CCACCTGCAAGACCATCGAC
		Reverse	CTGGCGAGCCTTAGTTTGGAC
Acan	Mouse	Forward	GTGGAGCCGTGTTTCCAAG
		Reverse	AGATGCTGTTGACTCGAACCT
Sox9	Mouse	Forward	AGTACCCGCATCTGCACAAC
		Reverse	ACGAAGGGTCTCTTCTCGCT
Col2a1	Mouse	Forward	GGGTCACAGAGGTTACCCAG
		Reverse	ACCAGGGGAACCACTCTCAC
CCL2	Mouse	Forward	TAAAAACCTGGATCGGAACCAAA
		Reverse	GCATTAGCTTCAGATTTACGGGT
CCL3	Mouse	Forward	TGTACCATGACACTCTGCAAC
		Reverse	CAACGATGAATTGGCGTGGAA
Ptges	Mouse	Forward	GGATGCGCTGAAACGTGGA
		Reverse	CAGGAATGAGTACACGAAGCC
MIF	Mouse	Forward	GAGGGGTTTCTGTCGGAGC
		Reverse	GTTCGTGCCGCTAAAAGTCA
HMBG1	Mouse	Forward	GCTGACAAGGCTCGTTATGAA
		Reverse	CCTTTGATTTTGGGGCGGTA
GADPH	Mouse	Forward	TTCCAGGAGCGAGACCCCACTA

		Reverse	GGGCGGAGATGATGACCCTTTT
Acan	Rat	Forward	AACTCAGTGGCCAAACATCC
		Reverse	TCAGGAATCCCAGATGTTCC
COL I	Rat	Forward	GAAGACCTGGCGAGAGAGGA
		Reverse	TCAATCCATCCAGACCGTTG
COL II	Rat	Forward	CTCAAGTCGCTGAACAACCA
		Reverse	GTCTCCGCTCTTCCACTCTG
Sox9	Rat	Forward	CTGAAGGGCTACGACTGGAC
		Reverse	TACTGGTCTGCCAGCTTCCT
GADPH	Rat	Forward	TTCCAGGAGCGAGACCCCACTA
		Reverse	GGGCGGAGATGATGACCCTTTT

Table S2. International Cartilage Repair Society macroscopic evaluation of cartilage repair (ICRS)

Categories	Score
**Degree of defect repair**	
In level with surrounding cartilage	4
75% repair of defect depth	3
50% repair of defect depth	2
25% repair of defect depth	1
No repair of defect depth	0
**Integration to border zone**	
Complete integration with surrounding cartilage	4
Demarcating border	3
Three-quarters of graft integrated, one-quarter with a notable border	2
One-half of graft integrated with surrounding cartilage, one-half with a notable border	1
From no contact to one-quarter of graft integrated with surrounding cartilage	0
**Macroscopic appearance**	
Intact smooth surface	4
Fibrillated surface	3
Small, scattered fissures or cracks	2
Several small or few large fissures	1
Total degeneration of grafted area	0
**Overall repair assessment**	
Grade I: normal	12
Grade II: nearly normal	8-11
Grade III: abnormal	4-7
Grade IV: severely abnormal	1-3

Table S3. The modified O’Driscoll histologic score (MODHS)

**Characteristic**	**Grading**	Score
I. % Hyaline cartilage	80–100	8
	60–80	6
	40–60	4
	20–40	2
	0–20	0
II. Structural characteristics		
A. Surface irregularity	Smooth and intact	2
	Fissures	1
	Severe disruption, fibrillation	0
B. Structural integrity	Normal	2
	Slight disruption, including cysts	1
	Severe lack of integration	0
C. Thickness	100% of normal adjacent cartilage	2
	50% to 100% or thicker than normal	1
	0–50%	0
D. Bonding to adjacent cartilage	Bonded at both ends of graft	2
	Bonded at one end/partially both ends	1
	Not bonded	0
III. Freedom from cellular changes of degeneration	Normal cellularity, no clusters	2
	Slight hypocellularity, <25% chondrocyte clusters	1
	Moderate hypocellularity, >25% clusters	0
IV. Freedom from degenerate changes in adjacent cartilage	Normal cellularity, no clusters, normal staining	3
	Normal cellularity, mild clusters, moderate staining	2
	Mild or mod hypocellularity, slight staining	1
	Severe hypocellularity, slight staining	0
V. Reconstitution of subchondral bone	Complete reconstitution	2
	Greater than 50% recon	1
	50% or less recon	0
VI. Bonding of repair cartilage to de *novosubchondral* bone	Complete and uninterrupted	2
	<100% but >50% recon	1
	<50% complete	0
VII. Safrinin O staining	>80% homogeneous positive stain	2
	40%–80% homogeneous positive stain	1
	<40% homogeneous positive stain	0
	Total score	Max27

References

1. Yin H, Wang Y, Sun Z, Sun X, Xu Y, Li P, et al. Induction of mesenchymal stem cell chondrogenic differentiation and functional cartilage microtissue formation for in vivo cartilage regeneration by cartilage extracellular matrix-derived particles. Acta Biomater. 2016; 33: 96-109.
